# Preventive Effects of a Kampo Medicine, Kakkonto, on Inflammatory Responses via the Suppression of Extracellular Signal-Regulated Kinase Phosphorylation in Lipopolysaccharide-Treated Human Gingival Fibroblasts

**DOI:** 10.1155/2014/784019

**Published:** 2014-02-18

**Authors:** Hiroyuki Kitamura, Hiroko Urano, Toshiaki Ara

**Affiliations:** ^1^Department of Hard Tissue Research, Graduate School of Oral Medicine, Matsumoto Dental University, Shiojiri, Nagano 399-0781, Japan; ^2^Institute for Oral Science, Graduate School of Oral Medicine, Matsumoto Dental University, Shiojiri, Nagano 399-0781, Japan; ^3^Department of Pharmacology, Matsumoto Dental University, 1780 Gobara, Hirooka, Shiojiri, Nagano 399-0781, Japan

## Abstract

Periodontal disease is accompanied by inflammation of the gingiva and destruction of periodontal tissues, leading to alveolar bone loss in severe clinical cases. The chemical mediator prostaglandin E_2_ (PGE_2_) and cytokines such as interleukin- (IL-)6 and IL-8 have been known to play important roles in inflammatory responses and tissue degradation. In the present study, we investigated the effects of a kampo medicine, kakkonto (TJ-1), on the production of prostaglandin E_2_ (PGE_2_), IL-6, and IL-8 by human gingival fibroblasts (HGFs) treated with lipopolysaccharide (LPS) from *Porphyromonas gingivalis*. Kakkonto concentration dependently suppressed LPS-induced PGE_2_ production but did not alter basal PGE_2_ levels. In contrast, kakkonto significantly increased LPS-induced IL-6 and IL-8 production. Kakkonto decreased cyclooxygenase- (COX-)1 activity to approximately 70% at 1 mg/mL but did not affect COX-2 activity. Kakkonto did not affect cytoplasmic phospholipase A_2_ (cPLA_2_), annexin1, or LPS-induced COX-2 expression. Kakkonto suppressed LPS-induced extracellular signal-regulated kinase (ERK) phosphorylation, which is known to lead to ERK activation and cPLA_2_ phosphorylation. These results suggest that kakkonto decreased PGE_2_ production by inhibition of ERK phosphorylation which leads to inhibition of cPLA_2_ phosphorylation and its activation. Therefore, kakkonto may be useful to improve gingival inflammation in periodontal disease.

## 1. Introduction

Periodontal disease is accompanied by inflammation of the gingiva and destruction of periodontal tissues, leading to alveolar bone loss in severe clinical cases. Prostaglandin E_2_ (PGE_2_), interleukin- (IL-)6, and IL-8 are known to play important roles in inflammatory responses and tissue degradation. PGE_2_ has several functions in vasodilation, the enhancement of vascular permeability and pain, and the induction of osteoclastogenesis and is believed to play important roles in inflammatory responses and alveolar bone resorption in periodontal disease [1]. IL-6 has the ability to induce osteoclastogenesis [2, 3]. IL-8 acts as a chemoattractant for neutrophils [4] that produce proteases such as cathepsin, elastase, and matrix metalloproteinase- (MMP-)8, leading to tissue degradation.

Recently, we reported that several kampo medicines, shosaikoto [5], hangeshashinto [6], and orento [7], suppress lipopolysaccharide- (LPS-) induced PGE_2_ production by HGFs and suggested that these kampo medicines have anti-inflammatory effects in periodontal disease. Another kampo medicine, kakkonto (TJ-1), has been clinically used for various diseases such as the common cold, coryza, the initial stage of febrile diseases, and inflammatory diseases. There are several reports that kakkonto shows antiallergic effects [8, 9] and antiviral effects [10–13] in animal and *in vitro* experimental models. For anti-inflammatory effects, kakkonto has been reported to decrease PGE_2_ production in cultured rabbit astrocytes [14]. Therefore, we considered the possibility that kakkonto decreases PGE_2_ production by human gingival fibroblasts (HGFs) and has anti-inflammatory effects with respect to periodontal disease. However, the anti-inflammatory effects of kakkonto are not adequately understood.

HGFs are the most prominent cells in periodontal tissue. Moreover, LPS-treated HGFs produce inflammatory chemical mediators such as PGE_2_ and inflammatory cytokines such as IL-6 and IL-8 [2, 15, 16]. Moreover, because HGFs have sustained production of PGE_2_ [17], IL-6, and IL-8 [18] in the presence of LPS, these mediators and cytokines in periodontal tissues are thought to be derived from HGFs. Therefore, we believe that examining the effects of drugs on HGFs, as well as on monocytes and macrophages, is important in the study of periodontal disease. In the present study, we examined the effect of kakkonto on LPS-induced PGE_2_, IL-6, and IL-8 production using this *in vitro* model.

## 2. Materials and Methods

### 2.1. Reagents

Kakkonto was purchased from Tsumura & Co. (Tokyo, Japan; lot number: D23122), and its components are listed in Table 1. Kakkonto was suspended in Dulbecco's modified Eagle's medium (D-MEM, Sigma, St. Louis, MO, USA) containing 10% heat-inactivated fetal calf serum, 100 units/mL penicillin, and 100 mg/mL streptomycin (culture medium) and was rotated at 4°C overnight. Then, the suspension was centrifuged and the supernatant was filtrated through a 0.45 *μ*m-pore membrane. Phorbol 12-myristate 13-acetate (PMA) purchased from Sigma. Other reagents were purchased from Nacalai tesque (Kyoto, Japan). LPS from *Porphyromonas gingivalis* 381 was provided by Professor Nobuhiro Hanada (School of Dental Medicine, Tsurumi University, Japan).

### 2.2. Cells

HGFs were prepared as described previously [6]. In brief, HGFs were prepared from free gingiva during the extraction of an impacted tooth with the informed consent of the subjects who consulted Matsumoto Dental University Hospital. The free gingival tissues were cut into pieces and seeded onto 24-well plates (AGC Techno Glass Co., Chiba, Japan). HGFs were maintained in culture medium at 37°C in a humidified atmosphere of 5% CO. For passage, HGFs were trypsinized, suspended, and plated into new cultures in a 1 : 3 dilution ratio. HGFs were used between the 10th and 15th passages in the assays. This study was approved by the Ethical Committee of Matsumoto Dental University (number 0063).

### 2.3. Cell Viability

The numbers of cells were measured using WST-8 (Cell Counting Kit-8; Dojindo, Kumamoto, Japan) according to the manufacturer's instructions. In brief, HGFs (10,000 cells/well) were seeded in 96-well plates (AGC Techno Glass Co., Chiba, Japan) and incubated in serum-containing medium at 37°C overnight. Then, the cells were treated with various concentrations of kakkonto (0, 0.5, 1, 2, 5, and 10 mg/mL) in the absence or presence of LPS (10 ng/mL) for 24 h (200 *μ*L each well) in quadruplicate for each sample. Then, the media were removed by aspiration and the cells were treated with 100 *μ*L of mixture of WST-8 with culture medium for 2 h at 37°C in CO incubator. Optical density was measured (measured wavelength at 450 nm and reference wavelength at 655 nm) using an iMark microplate reader (Bio-Rad, Hercules, CA, USA), and the mean background value was subtracted from each value. Data is represented as means ± SD (*n* = 4).

### 2.4. Enzyme-Linked Immunosorbent Assay (ELISA)

HGFs (10,000 cells/well) were seeded in 96-well plates and incubated in serum-containing medium at 37°C overnight. Then, the cells were treated with various concentrations of kakkonto (0, 0.01, 0.03, 0.1, 0.3, and 1 mg/mL) in the absence or presence of LPS (10 ng/mL) for 24 h (200 *μ*L each well) in triplicate for each sample. After the culture supernatants were collected, viable cell numbers were measured using WST-8 as described above. The concentrations of PGE_2_, IL-6, and IL-8 in the culture supernatants were measured by ELISA according to the manufacturer's instructions (PGE_2_, Cayman Chemical, Ann Arbor, MI, USA; IL-6 and IL-8, Biosource International Inc., Camarillo, CA, USA) and were adjusted by the number of viable cells. Data are represented as ng or pg per 10,000 cells (mean ± SD, *n* = 3).

### 2.5. Cyclooxygenase Activity

The effects of kakkonto on the activities of cyclooxygenase (COX)-1 and COX-2 were analyzed using a COX inhibitor screening assay kit (Cayman Chemical, Ann Arbor, MI, USA) according to the manufacturer's instructions. COX activities were evaluated by the measurement of prostaglandin produced from arachidonic acid by COX-1 or COX-2. These values were normalized to a relative value of 100% for cells without LPS or kakkonto treatments, and are represented as means ± SD (*n* = 3).

### 2.6. Western Blotting

HGFs were cultured in 60 mm dishes and treated with combinations of LPS and kakkonto for the indicated times. Then, cells were washed twice with Tris-buffered saline, transferred into microcentrifuge tubes, and centrifuged at 6,000 ×g for 5 min at 4°C. Supernatants were aspirated and cells were lysed on ice in lysis buffer (50 mM Tris-HCl, pH 7.4, 1% Nonidet P-40, 0.25% sodium deoxycholate, 150 mM NaCl, 1 mM ethyleneglycol bis(2-aminoethylether) tetraacetic acid (EGTA), 1 mM sodium orthovanadate, 10 mM sodium fluoride, 1 mM phenylmethylsulfonyl fluoride, 10 *μ*g/mL aprotinin, 5 *μ*g/mL leupeptin, and 1 *μ*g/mL pepstatin) for 30 min at 4°C. Then, samples were centrifuged at 12,000 ×gfor 15 min at 4°C, and supernatants were collected. The protein concentration was measured using a BCA Protein Assay Reagent Kit (Pierce Chemical Co., Rockford, IL, USA).

The samples (10 *μ*g of protein) were fractionated in a polyacrylamide gel under reducing conditions and transferred onto a polyvinylidene difluoride (PVDF) membrane (Hybond-P; GE Healthcare, Uppsala, Sweden). The membranes were blocked with 5% ovalbumin for 1 h at room temperature and incubated with primary antibody for an additional 1 h. The membranes were further incubated with horseradish peroxidase-conjugated secondary antibodies for 1 h at room temperature. Protein bands were visualized with an ECL Kit (GE Healthcare).

Antibodies against COX-2 (sc-1745, 1 : 500 dilution), cytoplasmic phospholipase A_2_ (cPLA_2_, sc-438, 1 : 200 dilution), annexin1 (sc-11387, 1 : 1,000 dilution), and actin (sc-1616, 1 : 1,000 dilution), which detects a broad range of actin isoforms, were purchased from Santa Cruz Biotechnology (Santa Cruz, CA, USA). Antibodies against extracellular signal-regulated kinase (ERK; p44/42 MAP kinase antibody, 1 : 1,000 dilution) and phosphorylated ERK (Phospho-p44/42 MAPK (Thr202/Tyr204) (E10) monoclonal antibody, 1 : 2,000 dilution) were from Cell Signaling Technology (Danvers, MA, USA). Horseradish peroxidase-conjugated anti-goat IgG (sc-2020, 1 : 20,000 dilution) was from Santa Cruz, and anti-rabbit IgG (1 : 20,000 dilution) and anti-mouse IgG (1 : 20,000 dilution) were from DakoCytomation (Glostrup, Denmark).

### 2.7. Statistical Analysis

**Figure 1:**
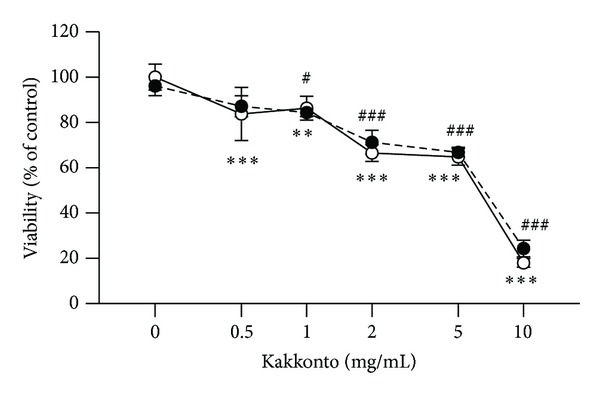
Effects of kakkonto on HGFs viability. The effect of kakkonto on the viability of HGFs at 24 h. HGFs were plated in 96-well microplates at 10,000 cells/mL, and media containing LPS and kakkonto were added. Cell numbers were evaluated by WST-8 at 24 h. The optical density (OD) was normalized to a relative value of 100% for cells without LPS or kakkonto treatments and is represented as means ± SD (*n* = 4). Open circles, treatment without LPS; closed circles, treatment with 10 ng/mL of LPS. **P < 0.01 and ***P < 0.001 (without kakkonto versus with kakkonto in the absence of LPS). ^#^ *P* < 0.01 and ^##^ *P* < 0.001 (without kakkonto versus with kakkonto in the presence of LPS). *P* values were calculated by pairwise comparisons and corrected with the Holm method (16 null hypotheses).

**Figure 2:**
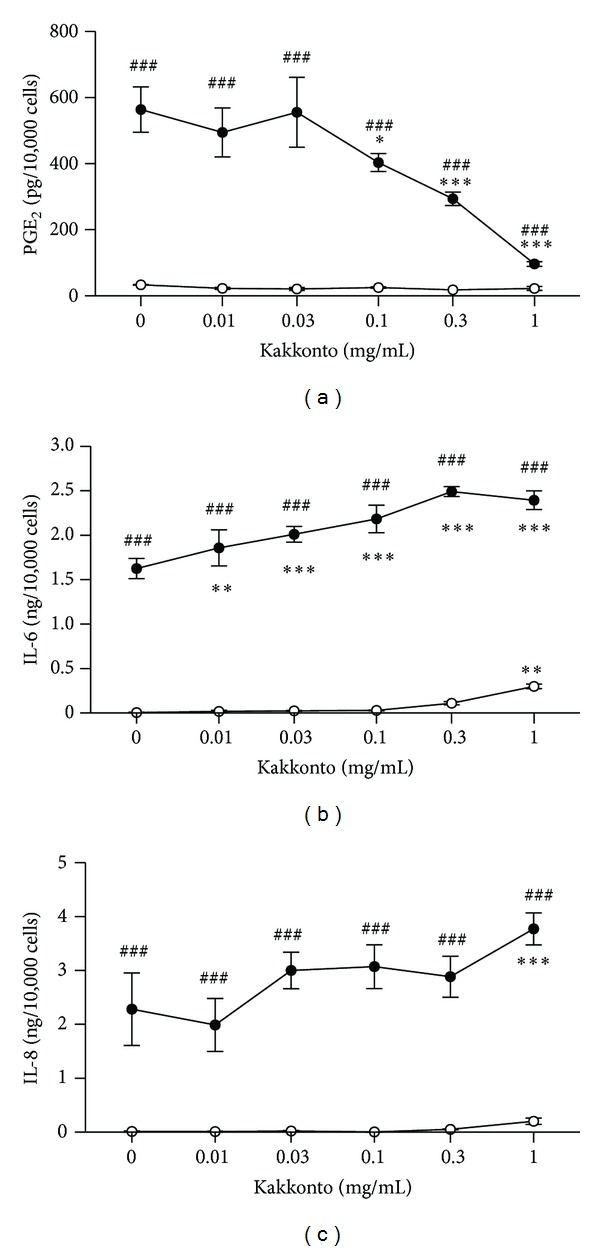
Effects of kakkonto on the production of PGE_2_, IL-6, and IL-8. HGFs were treated with combinations of LPS (0 and 10 ng/mL) and kakkonto (0, 0.01, 0.3, 0.1, 0.3, and 1 mg/mL) for 24 h. Concentrations of PGE_2_ (a), IL-6 (b), and IL-8 (c) were measured by ELISA, adjusted by cell number, and expressed as per 10,000 cells (mean ± SD, *n* = 3). Open circles, treatment without LPS; closed circles, treatment with 10 ng/mL of LPS. **P < 0.01 and ***P < 0.001 (without kakkonto versus with kakkonto). ^##^ *P* < 0.001 (without LPS versus with LPS). *P* values were calculated by pairwise comparisons and corrected with the Holm method (16 null hypotheses).

**Figure 3:**
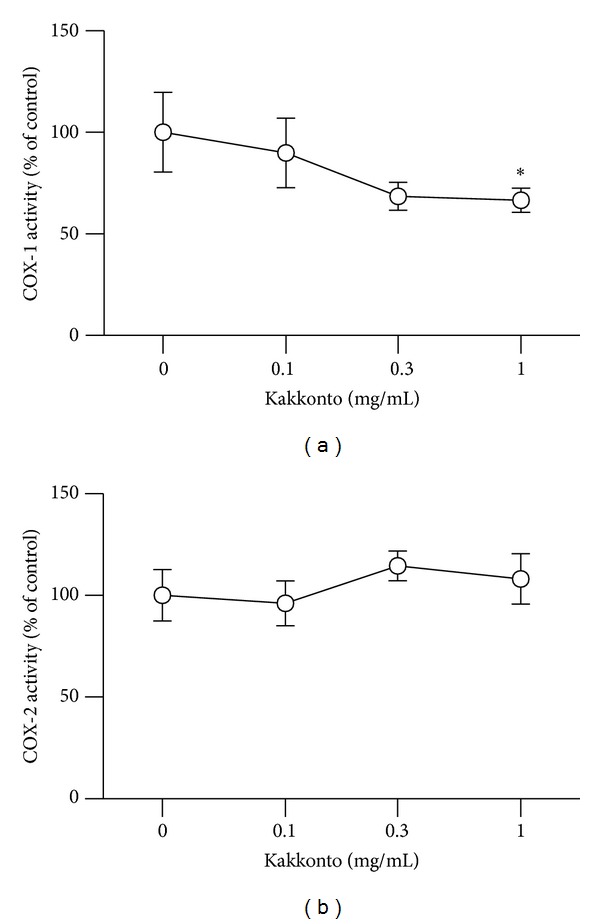
Effects of kakkonto on COX activities. COX activities were evaluated by measurement of prostaglandin produced from arachidonic acid by COX-1 or COX-2. These values were normalized to a relative value of 100% for cells without LPS or kakkonto treatments and are represented as means ± SD (*n* = 3). *P < 0.05 (Dunnett's test).

Differences between groups were evaluated by the two-tailed pairwise comparison test with a pooled variance, followed by correction with the Holm method (total 16 null hypotheses; 5 null hypotheses without kakkonto versus with kakkonto in the absence of LPS, 5 null hypotheses without kakkonto versus with kakkonto in the presence of LPS, and 6 null hypotheses without LPS versus with LPS) (Figures 1 and 2). Differences between the control group and experimental groups were evaluated by a two-tailed Dunnett's test (Figure 3).

All computations were performed with the statistical program R (http://www.r-project.org/). Dunnett's test was performed using the “glht” function in the “multcomp” package. Values with *P* < 0.05 were considered significantly different.

## 3. Results

### 3.1. Effects of Kakkonto on HGFs Viability

First, we examined the effect of kakkonto on HGFs viability. The viability of HGFs was approximately 90% at up to 1 mg/mL of kakkonto for a 24 h treatment in the absence or presence of LPS (Figure 1). The viabilities were approximately 70% and 20% at 5 mg/mL and 10 mg/mL of kakkonto, respectively (Figure 1). Therefore, we used kakkonto at the concentrations of up to 1 mg/mL in further experiments.

### 3.2. Effects of Kakkonto on PGE_2_, IL-6, and IL-8 Production

We examined whether kakkonto affects the production of PGE_2_ and inflammatory cytokines (IL-6 and IL-8) by HGFs. Because kakkonto affects cell viability, the concentrations of PGE_2_, IL-6, and IL-8 needed to be adjusted according to viable cell number.

When HGFs were treated with 10 ng/mL of LPS, HGFs produced large amounts of PGE_2_, IL-6, and IL-8. Indomethacin decreased LPS-induced PGE_2_ production in a concentration-dependent manner but slightly decreased LPS-induced IL-6 and IL-8 production (data not shown). Kakkonto significantly decreased PGE_2_ production in a concentration-dependent manner (Figure 2(a)). In the absence of LPS, kakkonto had no effect on PGE_2_ production (Figure 2(a)). In contrast, kakkonto increased LPS-induced IL-6 and IL-8 production (Figures 2(b) and 2(c)). In the absence of LPS, up to 0.1 mg/mL of kakkonto did not affect IL-6 and IL-8 production, but above 0.3 mg/mL of kakkonto, their concentrations were increased (Figures 2(b) and 2(c)). Similar results were obtained using human skin fibroblast TIG-103 cells (data not shown).

### 3.3. Effects of Kakkonto on COX Activities

Because PGE_2_ production is regulated by COX enzymes and suppressed by acid NSAIDs such as aspirin and diclofenac sodium, which inhibit COX activities, we examined whether kakkonto inhibits COX-1 and COX-2 activities. Kakkonto decreased COX-1 activity to approximately 70% at 1 mg/mL but did not affect COX-2 activity (Figure 3).

### 3.4. Effects of Kakkonto on Molecular Expression in the Arachidonic Acid Cascade

**Figure 4:**
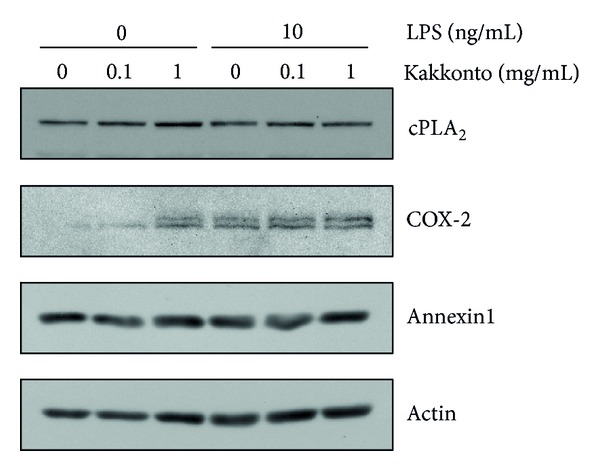
Effects of kakkonto on cPLA_2_, COX-2, and annexin1 expressions. HGFs were treated with a combination of LPS (0 or 10 ng/mL) and kakkonto (0, 0.01, or 1 mg/mL) for 8 h, and protein levels were examined by western blotting.

We examined whether kakkonto affects the expression of molecules in the arachidonic acid cascade. cPLA_2_ is the most upstream enzyme in the arachidonic acid cascade and releases arachidonic acid from plasma membranes. Kakkonto did not alter cPLA_2_ expression in the absence or presence of LPS (Figure 4). COX-2 was not detected in the absence of LPS. Treatment with kakkonto alone increased COX-2 expression. However, kakkonto did not alter LPS-induced COX-2 expression (Figure 4). Annexin1, also named lipocortin1, is an anti-inflammatory mediator produced by glucocorticoids that inhibit cPLA_2_ activity [19, 20]. However, neither LPS nor kakkonto showed an effect on annexin1 expression (Figure 4).

### 3.5. Effects of Kakkonto on ERK Phosphorylation

**Figure 5:**
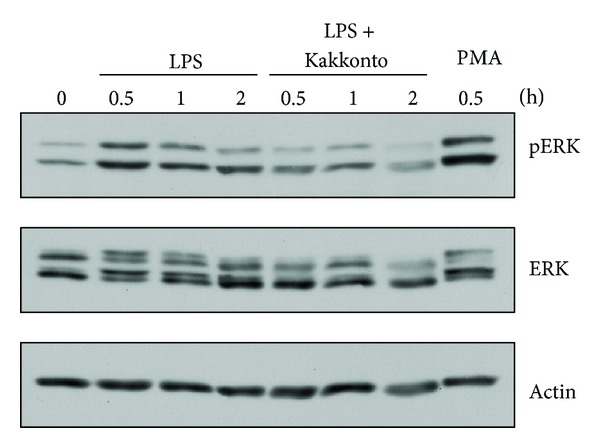
Effects of kakkonto on LPS-induced ERK phosphorylation. HGFs were untreated (0 h), treated with LPS (10 ng/mL), or treated with both LPS and kakkonto (1 mg/mL) for 0.5, 1, and 2 h. As a positive control, HGFs were treated with 1 *μ*M of PMA for 0.5 h. Western blotting was performed using antiphosphorylated ERK or anti-ERK antibodies. pERK: phosphorylated ERK. Upper band indicates ERK1 (p44 MAPK) and lower band indicates ERK2 (p42 MAPK).

cPLA_2_ is reported to be directly phosphorylated at Ser505 by ERK, resulting in cPLA_2_ activation [21, 22]. Therefore, we examined whether kakkonto suppresses LPS-induced ERK phosphorylation. ERK phosphorylation was enhanced at 0.5 h after LPS treatment and thereafter was attenuated. One mg/mL of kakkonto suppressed LPS-induced ERK phosphorylation at 0.5 h to 2 h (Figure 5).

## 4. Discussion

In the present study, we examined the effect of kakkonto on LPS-induced PGE_2_, IL-6, and IL-8 production by HGFs. Kakkonto concentration dependently decreased LPS-induced PGE_2_ production but did not affect PGE_2_ production without LPS treatment, similar to shosaikoto, hangeshashinto, and orento [5–7]. Moreover, kakkonto suppressed LPS-induced ERK phosphorylation. In contrast, kakkonto increased LPS-induced IL-6 and IL-8 production. It is widely known that PGE_2_ leads to inflammatory responses such as vasodilation, enhanced vascular permeability, and pain generation [1]. Acid non-steroidal anti-inflammatory drugs NSAIDs show anti-inflammatory effects by suppression of PGE_2_ production, even though they do not affect IL-6 and IL-8 production. Our findings showing that kakkonto decreases LPS-induced PGE_2_ production suggest that kakkonto also has anti-inflammatory effects in periodontal disease and that its effects are mainly mediated by suppression of PGE_2_ production even though kakkonto increased LPS-induced IL-6 and IL-8 production.

Our results showed that kakkonto suppressed LPS-induced ERK phosphorylation in HGFs. Previously, we demonstrated that orento inhibits LPS-induced ERK phosphorylation and cPLA_2_ activation, leading to the suppression of PGE_2_ production in HGFs [7]. Therefore, we consider that kakkonto decreased LPS-induced PGE_2_ production through the suppression of ERK phosphorylation in HGFs.

Although kakkonto increased COX-2 expression in the absence of LPS, kakkonto did not alter PGE_2_ production. We consider a likely reason to be the suppression of cPLA_2_ activation through the inhibition of ERK phosphorylation and/or the suppression of COX-1 activity. However, the components that induce COX-2 expression remain unknown.

Our results showed that kakkonto increased LPS-induced IL-6 and IL-8 production by HGFs. Previously, we reported that the activation of the protein kinase A (PKA) pathway by adrenaline or aminophylline increases LPS-induced IL-6 and IL-8 production in HGFs [23] and that H-89, a PKA inhibitor, decreases LPS-induced IL-6 and IL-8 production [23, 24]. Therefore, kakkonto may activate the PKA pathway.

In general, steroidal anti-inflammatory drugs (SAIDs) suppress the expression of cPLA_2_, COX-2, and inflammatory cytokines (such as IL-6 and IL-8) and induce the expression of annexin1. However, kakkonto did not affect cPLA_2_, annexin1, or LPS-induced COX-2 expression, and it increased IL-6 and IL-8 production. This therefore suggests that the mechanism by which kakkonto decreases PGE_2_ production is different from that of SAIDs.

Many studies have demonstrated that NSAID administration prevents gingival inflammation [25] and several clinical studies have indicated that the concentration of PGE_2_ in gingival crevicular fluid (GCF) is increased in periodontal disease [26] and is decreased by oral administration or mouthwash with NSAIDs [27, 28]. Considering that both NSAIDs and kakkonto suppress PGE_2_ production, it is possible that administration of kakkonto also decreases the PGE_2_ concentration in GCF and results in the improvement of gingival inflammation. Therefore, kakkonto may be useful for the improvement of gingival inflammation in periodontal disease. Importantly, kakkonto did not affect the basal level of PGE_2_, although kakkonto decreased COX-1 activity to approximately 70%. Because PGE_2_ produced by COX-1 protects gastric mucosa, these results suggest that kakkonto may cause minimal gastrointestinal dysfunction.

## 5. Conclusion

We demonstrated that kakkonto suppresses LPS-induced ERK phosphorylation, resulting in the suppression cPLA_2_ activation and further PGE_2_ production by HGFs. These results suggest that kakkonto is clinically useful for the improvement of inflammatory responses in periodontal disease.

## Figures and Tables

**Table 1:** Ingredients of the kakkonto formula.

Japanese name	Latin name	Amount (g)	Amount* (g/g of product)
Kakkon	*Puerariae Radix *	4.0	0.111
Taiso	*Zizyphi Fructus *	3.0	0.083
Mao	*Ephedrae Herba *	3.0	0.083
Kanzo	*Glycyrrhizae Radix *	2.0	0.056
Keihi	*Cinnamomi Cortex *	2.0	0.056
Shakuyaku	*Paeoniae Radix *	2.0	0.056
Shokyo	*Zingiberis Rhizoma *	2.0	0.056

Total		18.0	0.500

*7.5 g of kakkonto product contains 3.75 g of a dried extract of the mixed crude drugs.
